# Consensus of Return-to-Play Criteria After Adductor Longus Injury in Professional Soccer

**DOI:** 10.3390/sports13050134

**Published:** 2025-04-27

**Authors:** José Luis Estévez Rodríguez, Jesús Rivilla García, Sergio L. Jiménez-Sáiz, Sergio Jiménez-Rubio

**Affiliations:** 1 Switzerland National Team, Worbstrasse 48, 3074 Muri bei Bern, Switzerland; joselu.estevez@gmail.com; 2 Faculty of Physical Activity and Sport Sciences (INEF-Sports Department), Polytechnic University of Madrid, 28040 Madrid, Spain; jesus.rivilla@upm.es; 3 Sports Science Research Studies, University Rey Juan Carlos, Fuenlabrada, 28943 Madrid, Spain; sergio.jimenez.saiz@urjc.es

**Keywords:** exit criteria, rehabilitation, return to performance, groin injuries, muscle injuries

## Abstract

Return to play (RTP) decision making in professional soccer is crucial for minimising re-injury risk, reducing financial burdens on clubs, and optimising player performance. Despite its significance, there is a lack of objective criteria and consensus on RTP for adductor longus injuries, one of the most common muscle injuries in soccer. The aim of the present consensus was to validate an RTP protocol based on clinical, functional, and performance criteria through expert evaluation. This study hypothesises that a validated RTP protocol for adductor longus injuries will enhance decision making, reduce re-injury rates, and improve player performance upon return. An observational survey was designed to validate an RTP protocol through an expert panel. A total of 63 injury-management professionals (strength and conditioning coaches, physiotherapists, doctors, and rehabilitation fitness coaches) with an average experience of 12.02 ± 6.87 years participated in validating a 20-criteria RTP protocol. The protocol, divided into clinical, functional, and performance criteria, was assessed using a 5-point Likert scale. Aiken’s V coefficient was calculated for content validity, with criteria validated if Aiken’s V ≥ 0.75. Out of 20 initial RTP criteria, 14 were validated by the expert panel, with Aiken’s V ranging from 0.77 to 0.94 (overall range: 0.61–0.98). Key validated criteria included pain on palpation, flexibility, imaging, athlete feedback, strength assessments, movement quality, pre-injury GPS data, and performance under simulated match conditions. Criteria such as the Copenhagen adduction exercise and specific agility tests were not validated. The expert-validated RTP protocol for adductor longus injuries provides a structured approach to decision making, potentially reducing re-injury risk, improving rehabilitation strategies, and enhancing player performance. These findings could be integrated into clinical sports-medicine practices to enhance rehabilitation effectiveness and RTP decisions in professional soccer.

## 1. Introduction

One of the major factors that impact the professional development and performance of the soccer player is injuries. The overall incidence of injuries in professional male soccer players was 8.1 injuries/1000 h of exposure [1]. The second most common muscle injury is that affecting the adductor [2]. In elite soccer, the season prevalence of acute adductor time-loss injuries is 14%, resulting in a mean (± SD; standard deviation) absence of 14 ± 24 days [2]. In epidemiological studies on elite European soccer, the re-injury rate over the first two months after returning from injury was reported to be 18% after acute adductor injuries [2].When an injury occurs, it is essential to return quickly and with the lowest risk of recurrence to mitigate the effects on individual and team performance, as well as the implication that injuries have on the club’s finances [1,3].

The decision to return to play (RTP) is a complex process based on the assessment of health and risk activity but is also influenced by the assessment of risk tolerance [4]. The combination of clinical, physiological, conditional, and performance information will be essential for optimal decision making. In addition, multidisciplinary and interdisciplinary work will be one of the keys to success, but it will be fundamental to establish criteria with solid scientific evidence due to the lack of clarity and consensus in the RTP process [5,6]. Nowadays, rehabilitation and reconditioning programmes are becoming more and more complete and holistic in their approach, seeking to address each of the phases of the injury, as well as each of the functions developed by the different professionals involved in the process [6,7].

In addition, we have found some studies related to criteria for RTP, based on the Delphi consensus, where the criteria for hamstring and groin pain injuries were established [4,8,9]. We also found a recent review on RTP criteria in hamstring injuries, where these were divided into three categories: clinical, strength, and performance criteria [8]. These were based on criteria related to functional, performance, strength, flexibility, pain, and player’s confidence. Strength assessment, performance analysis, and a sport-specific skills assessment, such as sprinting in hamstring injuries, were highly valued as criteria in RTP decision making [4,9]. 

However, we did not find specific and objective information on the exit criteria, nor a consensus in the RTP for acute adductor injuries. The aim of this study will be to validate a protocol, composed of clinical, functional, and performance-based criteria for RTP decision making for adductor longus injuries through a panel of experts. The methodology used differs from that used in the Delphi consensus [4,9], where consensus is achieved through different rounds of anonymous questionnaires. In addition, this research focused on the adductor longus injury.

## 2. Methods

### 2.1. Study Design

An observational survey was designed to determine RTP after adductor longus injury, consisting of 20 criteria. This protocol was validated anonymously by a panel of experts and the most relevant criteria were used to determine the RTP protocol.

### 2.2. Participants

**Figure 1:**
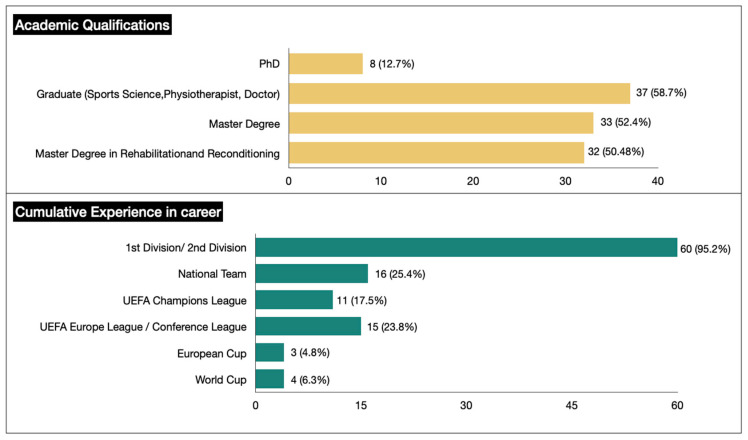
The profile of the panel of experts consisting of S&C coaches, physios, doctors and rehab fitness coaches.

A total of 67 experts in the field of injuries (S&C coaches, physios, doctors, and rehab fitness coaches) were contacted to validate the RTP protocol. Four experts did not respond to the calls or/and emails, and ultimately 63 participated (age = 37.85 ± 8.17 years) as experts in the validation. In addition, all experts signed a voluntary consent form where it was explained that they had the right to withdraw from the study. They had a professional experience of 12.02 ± 6.87 years in elite clubs and national teams (Figure 1). The nationality of experts were Spanish (*n* = 46), Swiss (*n* = 5), Portuguese (*n* = 2), Greek (*n* = 3), British (*n* = 1), Mexican (*n* = 1), Argentine (*n* = 1), American (*n* = 1), Italian (*n* = 2), and French (*n* = 1).

In order to be considered eligible to participate in the study, the following inclusion criteria were established: [1] Practical experience in rehabilitation programmes for at least 3 years; [2] experience in multidisciplinary and interdisciplinary work in elite and/or pre-elite soccer of at least 3 years; [3] experience in scientific methodology and/or clinical expertise. The entire study procedure was conducted in accordance with the outlines stated in the WMA Declaration of Helsinki on the ethical principles for research involving human subjects. The study was approved by the ethical committee of the Universidad Rey Juan Carlos (registration number: 1601202303423; 9 March 2023).

### 2.3. Procedure

The RTP questionnaire was composed of twenty criteria. The criteria were divided into three categories: clinical, functional, and performance criteria, all of which were selected based on the literature with the objective of investigating each expert’s assessment of the RTP process (Table 1).

The RTP questionnaire was submitted via email and collected using Google online forms. The sentence “Do you use/analyse X when evaluating RTP in adductor longus injury?” was used. It was a closed question, which had a unique response from the experts. They were asked to rate each criteria on a 5-point Likert-type scale to express their degree of agreement, with values from 1 to 5 (Strongly disagree (=1), disagree (=2), neutral (=3), agree (=4), Strongly agree (=5).

### 2.4. Statistical Analysis

The coefficient of content validation was calculated using Aiken’s V, and its 95% confidence intervals were also determined [10]. An item was determined to be validated when the value of Aiken’s V was ≥ 0.75 [10]. The calculations were carried out using Microsoft Excel 2016 software (Microsoft^®^, Redmond, WA, USA). 

## 3. Results

Of the 20 RTP criteria, only 14 were considered valid, as the quantitative values given by the group of 63 experts were very high and were reflected in the Aiken V values (Table 2).

## 4. Discussion

**Figure 2:**
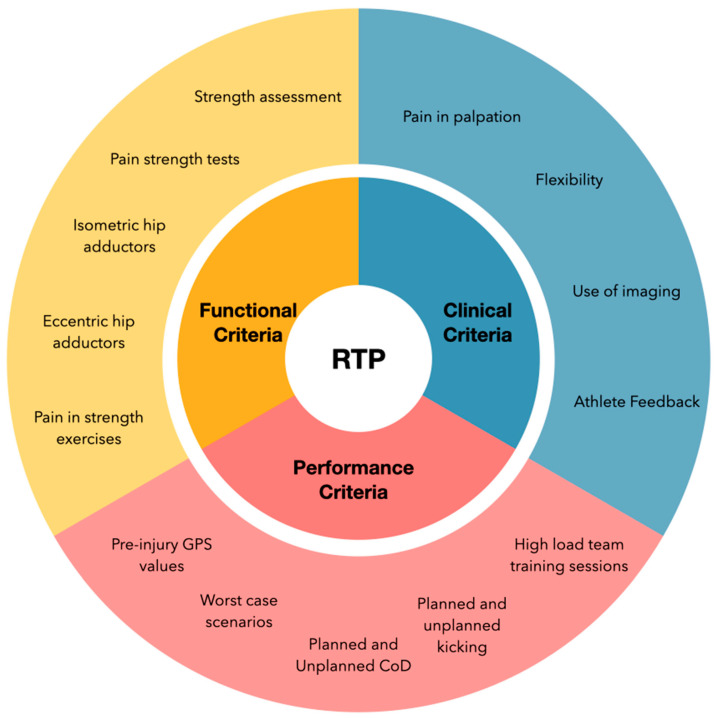
Validated RTP protocol. Only 14 criteria were determined as valid by the expert group.

The study was designed to the validation process of a protocol to improve decision-making during RTP. The protocol was composed of 20 criteria divided into three sections (clinical, functional, and performance). Sixty-three experts (S&C coaches, physios, doctors, and rehab fitness coaches) participated in the validation of the protocol, which proved to have high validity, with high scores obtained on each of the programme items, although it should be noted that 6 of the proposed criteria were not validated by the experts. In the end, 14 criteria were validated by the experts (Figure 2).

### 4.1. Clinical Criteria

Pain on palpation, flexibility, use of imaging techniques, and athlete’s feedback were highly rated (Aiken’s V = 0.75; 0.80; 0.77; and 0.94) by the experts. Recent studies indicate that palpation pain and flexibility during clinical examinations are of great importance during the injury recovery process, in order to progress through the different phases, but they are also of great relevance as criteria in the RTP. However, we must take these results with caution, as Delphi’s study [4] reported that there was no consensus among the participants regarding flexibility in adductor-related groin pain. On the other hand, previous studies related to RTP after hamstring injury observed flexibility as one of the most important criteria during RTP [11,12,13].

The criterion that received the highest value (Aiken’s V = 0.94) from the experts was athlete’s feedback. Previous studies showed the importance of this aspect in the RTP in muscle injuries [11,12,13]. Factors such as fear of pain, re-injury, and high motivation could have an important impact on the injured athlete and condition their RTP [8]. Moreover, the use of imaging was positively assessed by experts. It is a very useful tool to know the state of healing of the injured tissue and to have an objective assessment of the process from the clinical criterion, which can help us to identify red flags and to obtain a better diagnosis of the injury, but there are several studies that do not agree with establishing it as a criterion in the RTP [4,14,15]. As a downside, the HAGOS questionnaire was not validated by experts, although it is a very effective tool for identifying hip and/or groin problems [16], but this was perhaps insufficient to determine it as a criterion in the RTP (Aiken’s V < 0.62).

### 4.2. Functional Criteria

In relation to the functional criteria for determining the RTP, it should be noted that the experts valued very confidently those criteria related to tests to measure the levels of adduction/abduction strength, pain during the execution of these and different types of contraction. In a meta-analysis [17], the most relevant factors for determining athletes with hip/groin pain were determined. It was specified that the reduction in strength levels in the adductor squeeze test can be used for such determination. A cutoff point of 465.33 N was associated with a 72% increased risk of injury [18]. In addition, there is an 83% probability of injury when force to body mass are values below 6.971 N/kg. In conclusion, this test is a simple, reliable, and cost-effective screening tool [18]. Previous studies [4,19] as well as our results agree on the importance of assessing the isometric and eccentric strength of the adductors, and evaluating the symmetry between both sides, as it seems to be an important tool for the assessment of adductor isometric and eccentric strength.

On the other hand, it should be distinguished that jumping assessments such as the Counter-Movement Jump or the single-leg squat were not validated by the experts as RTP criteria (Aiken’s V < 0.70). Perhaps skills such as jumping or one-legged hopping are more closely related to knee injury, such as those affecting the ACL, as we have observed in previous studies [7,20]. Furthermore, a previous study [19] proposed the performance of 10 repetitions of the Copenhagen exercise as an RTP criterion, one of the most commonly used exercises during hip adductor injuries [21], but the experts did not validate this exercise for inclusion in the RTP protocol either (Aiken’s V < 0.67). This is perhaps an interesting tool to use during the rehabilitation process, but insufficient to assess the RTP after an adductor longus injury.

### 4.3. Performance Criteria

It should be noted that the experts validated with great importance the performance criteria, determining them to be probably the closest to the environment of the training and competition context [4,6,7,8]. The specific skills that were assessed by the experts were directly related to the mechanisms of adductor longus injury (kicking, sprint, and change in direction) [4,15,19]. It seems that “kicking a ball” or change in direction are actions that are directly related to adductor longus injury, so the assessment of these skills may serve as a more relevant criterion in the RTP assessment process [4]. Nevertheless, the experts did not consider the assessment of the sprint to be of great importance (Aiken’s V < 0.65), although previous studies [8,12] do justify the importance of this type of test in hamstring injury, probably because of its direct involvement in the mechanism of injury. On the other hand, they did not consider the manoeuvrability and/or agility tests (L-Test/T-Test or/and Illinois) to be of great relevance either, perhaps because they are carried out in a closed and planned environment. It should be distinguished that previous studies [22,23] have indicated the relevance of change in direction in hip/groin pain, as when an injury occurs there are kinematic and kinetic changes that can affect the correct mechanics of movement and thus increase the risk of re-injury, so the authors suggest the importance of working on the re-education of this skill during the rehabilitation process. The experts validated the evaluation of the change in direction in open and closed environments as a criterion, where uncertainty plays a fundamental role in the development of these tasks and brings the injured athlete closer to the real context of their sport [24,25].

In addition, the experts confidently valued the criteria related to the worst-case scenarios and the importance of reaching pre-injury GPS values (Aiken’s V > 0.80). Several authors [26,27] have already defined the importance of these aspects during the final phase of rehabilitation in injuries. A previous study [24] shows the “control-chaos continuum” that are used in the rehabilitation programmes to simulate situations of maximum demand close to the reality of competition. On the other hand, previous studies [6,7,24] defined the RTP criterion as achieving GPS performance values during the rehabilitation process that are close to pre-injury values, with the aim of adapting the injured athlete to the conditional demand that they will encounter on his return to training with the team and return to play. Finally, the return to team training and high-demand sessions with the team was rated very clearly by the experts. It will be the last step prior to the RTP and many studies [6,7,8,12,19,26,27] observed this phase of the process as a fundamental criterion for returning to competition.

To conclude, one of the future lines of research could be to evaluate the efficacy of the protocol in soccer players with an adductor longus injury. Additionally, it should be noted that the sample used was representative and that they had significant professional and academic experience, but the results should perhaps be interpreted with caution, and a larger sample and greater variability in the nationality of experts or the possibility that they can add comments or extra criteria could possibly be taken into account for a future line of research.

## 5. Conclusions

A protocol to improve decisions during RTP following an adductor longus injury was validated by a group of experts, although 6 criteria out of the 20 proposed were discarded. The protocol was divided into three categories in order to unify the RTP criteria of the different professionals involved in a rehabilitation programme. This protocol could help clubs in their decisions during a player’s return to competition after an adductor injury, in order to reduce the risk of re-injury, enhance the injured player’s performance, and reduce the costs generated by injury. 

## Figures and Tables

**Table 1:** RTP questionnaire.

Criteria	Question
Clinical criteria	-Do you analyse the presence of pain on palpation when evaluating RTP in adductor longus injury? -Do you analyse flexibility when assessing RTP in adductor longus injury? -Do you use imaging to evaluate RTP in adductor longus injury? -Do you use Hip and Groin Outcome Score (HAGOS) when evaluating RTP in adductor longus injury? -Do you analyse the athlete’s feedback during the RTP evaluation process in adductor longus injury?
Functional criteria	-Do you use strength testing when evaluating RTP in adductor longus injury? Hip adduction/abduction 45°/60°/90° (Ex: Force Frame Test Protocol) -Do you analyse the presence of pain on strength tests when evaluating RTP in adductor longus injury? -Do you use the pain-free Copenhagen adduction (10 repetitions) when assessing RTP in adductor longus injury? -Do you use isometric hip-adductor strength as a criterion for RTP in adductor longus injury? -Do you use eccentric hip-adductor strength as a criterion for RTP in adductor longus injury? -Do you analyse the presence of pain in basic strength exercises when evaluating RTP in adductor longus injury? -Do you use Counter-Movement Jump (CMJ) (presence of pain) and/or comparison with pre-injury value (Force Decks, apps or others) when evaluating RTP in adductor longus injury? -Do you use a side-to-side comparison in single-leg squat jump (Force Decks, apps or others) when evaluating RTP in adductor longus injury?
Performance criteria	-Do you analyse whether the injured athlete achieves 75–80% of pre-injury Global Positioning System (GPS) values when assessing RTP in adductor longus injury? -Do you use Repeated Sprint ability test when assessing RTP in adductor longus injury? -Do you use the L-Test/T-Test or/and Illinois agility test when evaluating RTP in adductor longus injury? -Do you use tasks in “worst case scenarios” (change in direction unplanned, sprints, High Chaos) when evaluating RTP in adductor longus injury? -Do you analyse quality of movement in planned and unplanned change in direction when evaluating RTP in adductor longus injury? -Do you use planned and unplanned kicking tasks at different distances and intensities when evaluating RTP in adductor longus injury? -Do you analyse whether the injured athlete achieves high-load team training sessions (3–5 sessions) when evaluating RTP in adductor longus injury?

**Table 2:** The item content relevance score for all the twenty items as per the 63 experts, the Aiken’s V and the 95% confidence intervals (CI) of Aiken’s V. The ratings of the 63 experts varied on a scale from 1 to 5, where 1 corresponded to very poor relevance and 5 corresponded to very high relevance.

Ítem	1	2	3	4	5	Average	Aiken’s V (95% CI)
1. Pain in palpation	1	4	7	33	18	4.00	0.75 (0.59–0.85)
2. Flexibility		1	10	28	24	4.19	0.80 (0.64–0.89)
3. Use of imaging		2	13	25	23	4.10	0.77 (0.62–0.87)
4. Hip and Groin Outcome Score	3	8	18	25	9	3.46	0.62 (0.46–0.74)
5. Athlete feedback				16	47	4.75	0.94 (0.81–0.97)
6. Strength assessment		1	5	27	30	4.37	0.84 (0.69–0.92)
7. Pain strength tests			1	21	41	4.63	0.91 (0.78–0.96)
8. Copenhagen adduction		4	24	23	12	3.68	0.67 (0.51–0.79)
9. Isometric hip adductors		1	7	31	24	4.24	0.81 (0.66–0.90)
10. Eccentric hip adductors	2	2	9	24	26	4.11	0.78 (0.62–0.87)
11. Pain in strength exercises		2	3	27	31	4.38	0.85 (0.70–0.92)
12. CMJ	1	6	17	18	21	3.83	0.71 (0.55–0.82)
13. Single-leg squat jump	1	7	16	21	18	3.76	0.69 (0.53–0.81)
14. Pre-injury GPS values	1	1	8	23	30	4.27	0.82 (0.67–0.90)
15. Repeated Sprint ability test		9	23	16	15	3.59	0.65 (0.49–0.77)
16. L-Test/T-Test or agility test	2	5	22	21	13	3.60	0.65 (0.49–0.77)
17. Worst-case scenarios	2	1	5	23	32	4.30	0.83 (0.68–0.91)
18. Planned and unplanned CoD		2	9	32	20	4.11	0.78 (0.62–0.87)
19. Planned and unplanned kicking			3	25	35	4.51	0.88 (0.74–0.94)
20. High-load team training sessions			9	27	27	4.29	0.82 (0.67–0.91)

## Data Availability

The original contributions presented in the study are included in the article; further inquiries can be directed to the authors.
